# Polygenic risk score for autoimmune disorder SLE predicts current depression but independently of exposure to recent stress

**DOI:** 10.1192/j.eurpsy.2025.608

**Published:** 2025-08-26

**Authors:** A. Berezvai, D. Torok, S. Krause, G. Bagdy, G. Juhasz, X. Gonda

**Affiliations:** 1 Department of Pharmacodynamics, Faculty of Pharmacy; 2 NAP3.0-SE Neuropsychopharmacology Research Group, Hungarian Brain Research Program; 3 Doctoral College, Pharmaceutical Sciences and Health Technologies Division; 4 Doctoral College, Mental Health Sciences Division; 5 Department of Psychiatry and Psychotherapy, Semmelweis University, Budapest, Hungary

## Abstract

**Introduction:**

Inflammation has emerged in recent year as one potential contributor pathway in the background of several neuropsychiatric disorders, including depression. It is also increasingly understood that inflammation may exert its role via mediating the effect of stress in the etiology of such conditions. Therefore, understanding the role of inflammation in the background of depression would offer novel insight into the involved processes facilitating the identification of related biomarkers, novel treatment targets, as well as possibilities for early screening, risk identification, and prevention and intervention. In spite of these promising possibilities, currently we know little about the shared genetic risk in case of autoimmune or inflammatory conditions and depression.

**Objectives:**

Our present analyses aimed at investigating, using polygenic risk scores, if genetic predisposition to inflammatory or autoimmune disorders predicts depression scores and if recent exposure to life stressors interacts with the effect of genetic variation.

**Methods:**

For establishing polygenic risk scores (PRS) for different inflammatory/autoimmune conditions, we pre-existing GWAS summary statistics were used for systemic lupus erythematosus (SLE) (7219 cases, 15991 controls), autoimmune thyroid disease (AT) (859 cases, 324074 controls), and atrophic gastritis (AG) (180 cases, 456168 controls). The NewMood database (N=1820) was used as target data. Brief Symptom Inventory Depression Scale scores reflecting current depressive symptom severity was used as outcome depression phenotype. PRS calculations were performed with LDpred2. Linear regression models performed with R (4.1.3) were used to investigate the main effect of the PRS and the interacting effect of recent stress and PRS on current depression score.

**Results:**

The main effect analyses showed that SLE-PRS had a significant effect on depression (beta=0.2389, p=0.0175) while AT-PRS (beta=0.4094, p=0.9763) and AG-PRS (beta=0.4741, p=0.5795) did not show significant main effects. Interaction analyses did not show significant effect in case of any of the examined autoimmune/inflammatory disorders (SLE-PRS x recent stress: beta=0.1041, p=0.2016; AT-PRS x recent stress: beta=19.82, p=0.05811; AG-PRS x recent stress: beta=0.3169, p=0.6661).

**Conclusions:**

Our present results show that while there is an overlap in the genetic background of depression and some autoimmune or inflammatory conditions, current stressors may not have an interacting role in this relationship. Our results need replication in larger samples, and should be extended to investigate polygenic scores for other conditions and other types of stress.

This study was funded by NAP2022-I-4/2022, K143391, 2019-2.1.7-ERA-NET-2020-00005, TKP2021-EGA-25, ÚNKP-23-3-I-SE-73.

**Disclosure of Interest:**

None Declared

